# The Early Symptoms of General Paralysis

**Published:** 1895-01-12

**Authors:** 


### THE EARLY SYMPTOMS OF GENERAL PARALYSIS.

Much has been said against the name which has been given to this disease. It has been said, truly enough, that in given phases of general paralysis of the insane, there need not be paralysis, there need not be insanity, and that if paralysis is present it may not be general, but take the form rather of a local paresis. All this is true enough in regard to snap shots, taken at odd moments, in the course of the disease; but sooner or later we get the typical association of general paralysis with insanity. One ceases to be surprised at the curious diversities and irregularities of the earlier symptoms, or at their drifting, as they all do, towards the same goal, when the post-mortem appearances are taken into consideration. The mixed hypertrophies and degenerations of which these consist are not confined to the brain, or to the spinal cord, or to the membranes of either; even the nerves are affected; and looking at these widespread degenerations one need not wonder that, in the early stages of the disease, the symptoms show considerable variety and the diagnosis is often difficult.

The symptoms of the disease may be thrown into three great groups, so that its history may be told either as that of a gradually increasing muscular paralysis; or of an insanity of varied type and degree, mostly showing great exaltation, but ending always, if time is given, in utter dementia; or, lastly, as that of a series of paroxysms of convulsion, apoplexy, or mania, each landing the patient, both muscularly and mentally, at a lower level; and it is obvious what a varied picture may be drawn according to the preponderance and rate of development of these elements in its construction.

To the physician the paralytic symptoms are, from a diagnostic point of view, of the greater interest, for when the characteristic mental symptoms are developed they give the clue to the diagnosis. The trouble is when the paralyses precede, or are accompanied by depression rather than exaltation. For in truth the paralyses by themselves may not always at first stand out very clearly from the early signs of ataxy, or peripheral neuritis, or the various spinal scleroses, by the intercurrence of which, in fact, they possibly are caused.

The paralytic symptoms generally show themselves first in the tongue, lips, or hands, and take the form of tremor. Even earlier there may be inequality of the pupils. The tremor occurs mostly on attempting to use the muscles, at first is of a fibrillary character, and is often accompanied by a hesitancy or incoordination at the commencement of speech. As the weakness progresses it becomes difficult to pronounce certain words, articulation is slurred, and not only so, but words are clipped or even omitted, and syllables are left out.

Weakness of the hand shows itself generally in the writing as early as in anything, unless it be in other things requiring special skill, such as playing on the violin. Here again not only is the writing apt to be slurred and tremulous, but, as in speech, words or syllables are apt to be omitted or words left unfinished.

The gait soon becomes tottering or uncertain, often from mere weakness, at other times from inco-ordination or spastic conditions. There is often considerable numbness, which later on increases so that in the developed disease there may be almost complete anæsthesia.

There is found then a curious mixture of the cord and nerve symptoms which occur in those diseases with which, in its very early stages, it is most apt to be confused, viz., locomotor ataxy, disseminated sclerosis, and peripheral neuritis from alcoholism or syphilis. It is to be noted, however, that in general paralysis the tremor is more fibrillary than in other forms, not a massive moving to and fro, but a trembling of the smaller fibres of the muscles, and that it is often associated with a jerking hesitancy or almost spasm at the beginning of a movement; still it is clear what an assistance in regard to diagnosis is the concurrence with these symptoms of those pointing to the characteristic mental change. There is said to be a prodromal stage, the man becoming careless, spending money over-freely, giving way to intemperance or sexual excess; but these symptoms, together with that loss of intellectual power which shows itself most markedly by failing memory, may well be looked upon rather as indicative of an early stage of the disease. At any rate, if such symptoms as these, especially if combined grandiose delusions show themselves in combination with such signs of tremor or paralysis as have already been mentioned, the diagnosis of general paralysis of the insane becomes strongly emphasised, and when, with growing weakness, the trembling at the corners of the eyes and mouth persists, even when at rest, it is clenched beyond the possibility of doubt. It must not be forgotten, however, that the delusions are not always grandiose.

It is in the early stages that the difficulty arises, and then for a considerable time things may seem very doubtful, till, perhaps, after a convulsive or apoplectic attack, followed may be by a temporary one-sided paralysis, the whole picture may quickly develop into certainty, or more gradually the association of the paralytic with the mental symptoms may declare the nature of the case.

